# Health economic evaluation of polytrauma treatment in the current aG-DRG system – Results of a Level I Trauma Center

**DOI:** 10.1007/s00068-026-03368-1

**Published:** 2026-10-23

**Authors:** Katja Hierl, Claudius Thiedemann, Maximilian Kerschbaum, Daniel Popp, Volker Alt

**Affiliations:** Department of Trauma Surgery, University Hospital Regensburg, Regensburg, Germany

**Keywords:** Multiple trauma, Injury Severity Score, Operation room, Intensive Care Unit, Reimbursement, Cost calculation

## Abstract

**Purpose:**

Resource-intensive polytrauma care is associated with high treatment and readiness costs and poses a financial challenge for hospitals. The aim of this retrospective single-center study of the years 2020 to 2022 was to analyze polytrauma reimbursements and costs.

**Methods:**

Based on billing and clinical data of 201 polytrauma cases from a German University Hospital and Level I Trauma Center, revenues and costs of the eleven MDC 21 A polytrauma DRGs were analyzed. Ratios of the revenue in relation to LOS, ventilation hours, OR time and implant costs as well as personnel costs were calculated. Contribution margins were determined as an upper-bound estimate.

**Results:**

DRG reimbursement was highest for the most severely injured patients (50,852 €, DRG W36Z). It was lowest for patients who died within five days (2,214 €, DRG W60Z) or without surgeries (4,056 €, DRG W61B). The contribution margin was also highest for the most severely injured patients (52,775 €, DRG W36Z). Revenues in relation to ICU LOS (3,455 €/day, DRG W36Z) and ventilation time (238 €/hour, DRG W01B) were lowest for the most severe polytraumas. Regarding OR time and implant costs, reimbursement was lowest for injuries of the spine, pelvis and extremities with 62 €/min (DRG W02B) and 5 €/€ (DRG W02A).

**Conclusion:**

Treatment of polytrauma patients with long ICU stay, extended mechanical ventilation and multiple injuries of the extremities, pelvis and spine is not adequately reflected by the current per-case reimbursement in relation to the resources used (ICU days, ventilation hours, OR time and implant costs).

**Supplementary Information:**

The online version contains supplementary material available at https://doi.org/10.1007/s00068-026-03368-1.

## Introduction

 Since the introduction of the G-DRG (German Diagnosis Related Groups) system in 2004 with per-case reimbursement payments, the economic aspects of polytrauma care have been discussed controversially. Within the TraumaNetzwerk DGU^®^, local, regional and supraregional trauma centers across Germany are interconnected to enable competent, quality-assured and interdisciplinary care for severely injured patients around the clock 365 days a year [1–3]. In case of transfers of multiple injured patients within the trauma networks, there may be certain billing conditions under G-DRG terms that the trauma centers should pay attention to [4].

In Germany, approximately 35,000 individuals per year sustain severe injuries, defined as an Injury Severity Score (ISS) ≥ 16, the majority of whom are of working age [5]. This can lead to a medical but also socioeconomic burden due to the high overall treatment costs [6–9]. The majority of the total costs for acute hospital treatments of severely injured patients are incurred in the intensive care unit (ICU) and operation room (OR) [10–12]. All fixed and variable costs such as implant or drug costs are reimbursed within the G-DRG flat-rate. For very special and costly interventions such as dialysis or extracorporal membrane oxygenation (ECMO), additional nationwide or hospital-specific charges are reimbursed.

In addition to the treatment costs, there are high readiness costs for the 24/7 availability of emergency room including shock room as well as operation and ICU capacities, which are primarily incurred in the specialized trauma centers to ensure an optimal polytrauma treatment [13–16]. The costs of maintaining the infrastructure must be borne by the hospitals regardless of its actual utilization and therefore represent a significant economic challenge. Different studies showed that the costliest proportion was clinical medical staff while prevention and educational programs represent the smallest cost components [17, 18]. Due to the high treatment efforts and the limited possibilities for cost savings, the care of multiple injured patients but also of extreme cost cases with multimorbidity and interdisciplinary treatments, long length of hospital stays or multiple surgical interventions can lead to a serious financial burden for the hospitals [19, 20]. Against this background, within the framework of the ongoing German health care reform, there are plans to introduce readiness payments in order to reduce the economic pressure on hospitals [21, 22].

Since polytrauma patients usually have different injuries and can be very heterogeneous from a medical and economic point of view, their adequate and effort-appropriate assigning and classification within a flat-rate payment system seems very difficult [23–25]. Different previous studies showed that treatment of severely injured patients is not cost-covering in the G-DRG system and that hospitals incurred losses due to insufficient per-case reimbursements [26–29]. Also in other countries, such as Switzerland, significant deficits in acute therapy of polytrauma patients were calculated for hospitals, depending on the patient`s insurance status [30]. However, there are reports of possible cost recovery and even excess coverage for the hospitals, based on continuous developments and improvements of the G-DRG system, such as introduction of OPS-code 8-98f.- for intensive medical complex treatment or classification into different polytrauma DRGs of the Major Diagnostic Category (MDC) 21 A in the diagnosis related groups catalog [31, 32]. Additionally, high-quality treatment of severely injured patients can lead to a reduction in costs and revenue deficits for the hospitals. Glance et al. showed that polytrauma care in hospitals with low risk-adjusted mortality rates is associated with lower costs compared to average-quality hospitals [33].

In the current literature, there is no data that directly compares revenues and costs of the eleven MDC 21 A polytrauma DRGs in the German payment system. Therefore, this study aims to analyze and compare reimbursements of polytrauma cases with different degrees of severity grouped into MDC 21 A for a German University Hospital and Level I Trauma Center to demonstrate the revenues with regard to different parameters such as length of hospital and ICU stay, ventilation and OR time as well as implant costs. Taking into account OR costs and personnel costs, the respective contribution margins per polytrauma DRG are to be determined approximately in order to illustrate current revenue structure for polytrauma care in the German healthcare system.

## Materials and methods

### Study design and data collection

Clinic-specific data of DRG-based selected polytrauma cases from a German University Hospital and Level I Trauma Center were retrospectively analyzed with comparison of MDC 21 A DRGs. The research project received prior approval from the ethics committee of the affiliated university (Nr. 23-3401-104).

**Fig. 1:**
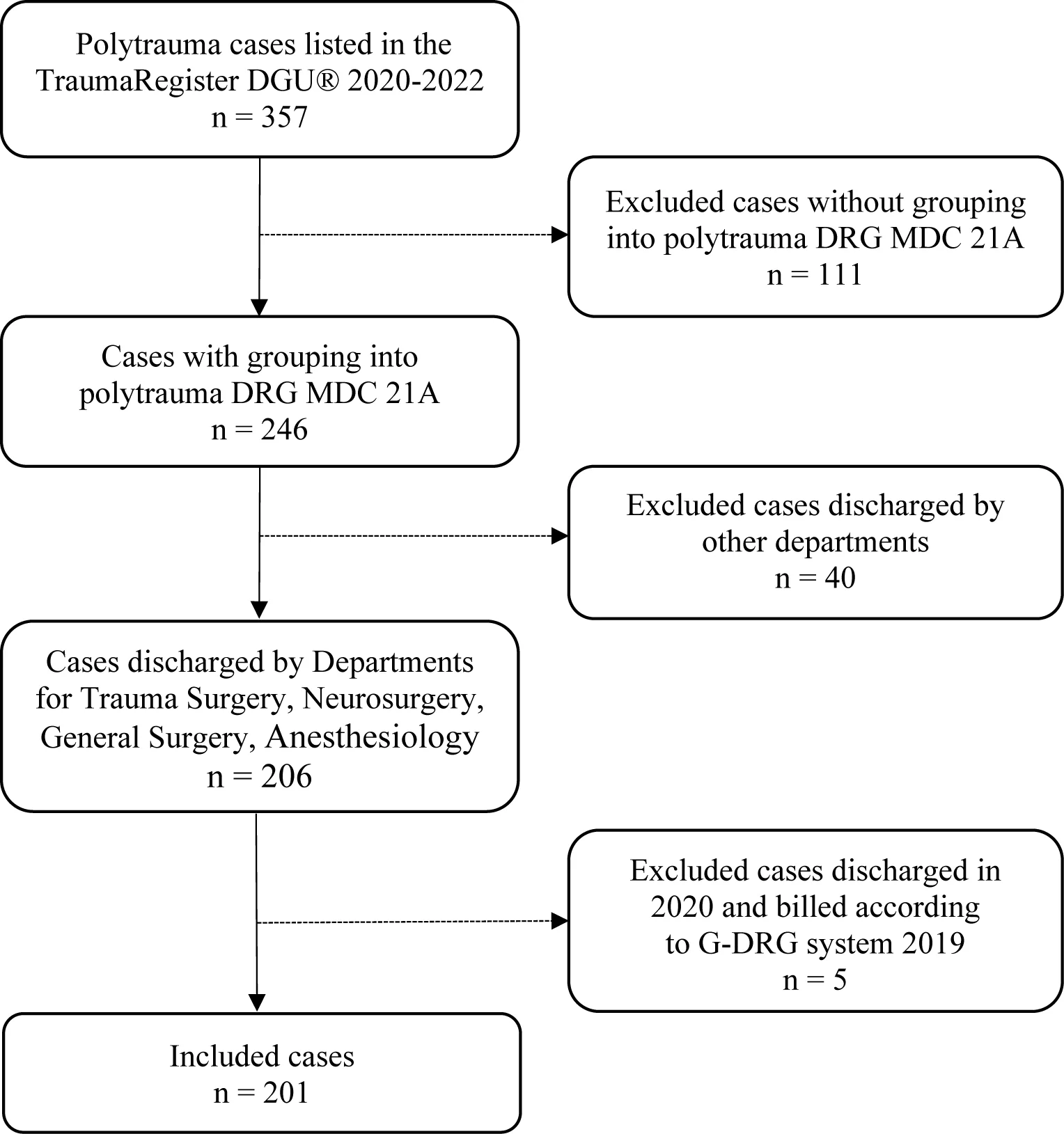
Case selection process. DRG diagnosis related groups; MDC major diagnostic category; n number

We collected the data of all polytrauma cases of the analyzed trauma center listed in the TraumaRegister DGU^®^ for the years 2020 to 2022 (*n* = 357). Of these, cases classified into polytrauma DRGs of MDC 21 A in the in-house data-set were included (*n* = 246). All cases of the departments leading in polytrauma care (Trauma Surgery, Neurosurgery, General Surgery, Anesthesiology) were included (*n* = 206). Cases discharged by other departments (Pediatrics, Plastic Surgery, Oral and Maxillofacial Surgery, Ear-Nose-Throat Medicine, Thoracic Surgery, Cardiovascular Surgery, Cardiology) were excluded (*n* = 40). Due to the reform of the remuneration system with nursing budget carve-out in 2020, cases with discharge in 2020 and billing according to the G-DRG system 2019 were excluded (*n* = 5). A total number of 201 cases was included (Fig. 1). The age criterion did not explicitly play a role in case selection.

Based on the DRG classification, polytrauma cases to be analyzed were extracted from the entire dataset of the University Hospital by the Medical Controlling Department. Clinical data and implant costs were collected from the hospital information system SAP IS-H (SAP SE, Walldorf, Germany) using medical reports as well as surgical and implant documents. The Purchasing and Logistics Department of the University Hospital provided cost data for special implants used in polytrauma surgery.

Based on the underlying DRG classification, following eleven groups according to MDC 21 A were compared (Table 1):

Group 1: DRG W01B

Group 2: DRG W01C

Group 3: DRG W02A

Group 4: DRG W02B

Group 5: DRG W04A

Group 6: DRG W04B

Group 7: DRG W04C

Group 8: DRG W36Z

Group 9: DRG W60Z

Group 10: DRG W61A

Group 11: DRG W61B

**Table 1:** Major Diagnostic Category (MDC) 21 A Polytrauma according to diagnosis related groups (DRG) catalog 2022

DRG	Partition	Designation	Cost Weight
W01B	O	Polytrauma with ventilation > 72 hours or complex procedures or IntK > 392 / 368 / 552 effort points, without early rehabilitation, with ventilation > 263 hours or with complex vacuum treatment or with IntK > 588 / 552 / - effort points	10.164
W01C	O	Polytrauma with ventilation > 72 hours or complex procedures or IntK > 392 / 368 / 552 effort points, without early rehabilitation, without ventilation > 263 hours, without complex vacuum treatment, without IntK > 588 / 552 / - effort points	4.319
W02A	O	Polytrauma with other complex procedures with complicating constellation or procedures at multiple locations or with intensive medical complex treatment > 392 / 368 / - effort points	7.999
W02B	O	Polytrauma with other complex procedures without complicating constellation, without procedures at multiple locations, without intensive medical complex treatment > 392 / 368 / - effort points	4.483
W04A	O	Polytrauma with other interventions or ventilation > 24 hours, with complicating constellation or interventions at multiple locations	5.422
W04B	O	Polytrauma with other procedures or ventilation > 24 hours, without complicating constellation, without procedures at multiple locations, with certain other procedures or ventilation more than 24 hours	3.168
W04C	O	Polytrauma with other procedures or ventilation > 24 hours, without complicating constellation, without procedures at multiple locations, without certain other procedures, without ventilation > 24 hours	2.360
W36Z	O	Intensive care complex treatment > 784 / 828 / - effort points for polytrauma or polytrauma with ventilation or craniotomy with endovascular implantation of stent prostheses in the aorta	12.111
W60Z	M	Polytrauma deceased < 5 days after admission, without complicating constellations, without ventilation > 24 hours, without complex or certain other procedures	0.533
W61A	M	Polytrauma without significant interventions with complicating diagnosis or with intensive medical complex treatment > 196 / 184 / - effort points or age < 12 years	1.654
W61B	M	Polytrauma without significant interventions, without complicating diagnosis, without intensive medical complex treatment > 196 / 184 / - effort points, age > 11 years	1.168

DRG diagnosis related groups; IntK intensive care complex treatment; M medical; O operative

Both groups 1–11 and the total collective of polytrauma cases were evaluated with regard to the following clinical, billing and cost data.

Clinical data:

- Gender (female/male)
- Age (years)
- ISS
- Total length of stay (LOS, days)
- LOS ICU (days)
- LOS peripheral ward (PW, days)
- Ventilation time (hours)
- OR time (= suture-incision time, minutes)

Billing and cost data:

- DRG.
- Patient Clinical Complexity Level (PCCL)
- Deduction (€)
- Surcharge (€)
- Cost Weight
- DRG revenue (€)
- Care revenue (€)
- Additional charge (€)
- Total revenue (€)
- Base rate (€)
- Care fee value (€)
- Implant costs (€)

For DRG classification of the treatment cases, grouper software 3M^™^ Kodip^®^ Suite (3 M Company, Neuss, Germany) versions 2020 to 2022 developed for the respective year was used. Both DRG reimbursements and nursing care reimbursements were calculated at the case level based on the respective state-specific base rate and preliminary or hospital-specific care fee value of each year. Since we calculated means and standard deviations from this using Excel 2019, revenues were not adjusted to a common reference. All calculated revenues exclude COVID-19-related compensation payments. Additional charges for coronavirus tests were not taken into account.

### State-specific base rate

2020: 3,660.92 €

2021: 3,739.35 €

2022: 3,825.44 €

### Care fee value

01.01.2020–31.03.2020: 146.55 € (Preliminary)

01.04.2020–31.12.2020: 185.00 € (COVID-19 pandemic regulation)

01.01.2021–31.08.2021: 163.09 € (Preliminary)

01.09.2021–31.12.2021: 375.61 € (Hospital-specific)

01.01.2022–31.08.2022: 199.33 € (Preliminary)

01.09.2022–31.12.2022: 440.20 € (Hospital-specific)

### Statistical evaluation

Data were collected pseudonymized by assigning patient identification and case numbers in tabular form using Excel 2019 (Microsoft Corp., Redmond, WA, USA). Mean values and standard deviations (SD) of all data were calculated by Excel 2019 as descriptive statistics to provide an overview of the extensive data. For clearly skewed variables (ventilation time, additional charge, implant costs) median and interquartile range were added using Excel 2019. Distribution was not assessed.

### Calculations

#### Ratios

To analyze revenue in relation to total LOS, LOS ICU, LOS peripheral ward, ventilation time, OR time and implant costs, six ratios were calculated as the ratio of the group mean values.

$$\begin{array}{ll}\text{Ratio 1} = \frac{\text{Total revenue (}\euro\mathrm{)}}{\text{Total LOS (days)}}&\text{Ratio 4} = \frac{\text{Total revenue (}\euro\mathrm{)}}{\text{Ventilation time (hours)}}\\[12pt]\text{Ratio 2} = \frac{\text{Total revenue (}\euro\mathrm{)}}{\text{ICU LOS (days)}}&\text{Ratio 5} = \frac{\text{DRG revenue (}\euro\mathrm{)}}{\text{OR time (minutes)}}\\[12pt]\text{Ratio 3} = \frac{\text{Total revenue (}\euro\mathrm{)}}{\text{Peripheral ward LOS (days)}}&\text{Ratio 6} = \frac{\text{DRG revenue (}\euro\mathrm{)}}{\text{Implant costs (}\euro\mathrm{)}}\end{array}$$

#### Personnel costs

We used a health-economic model to calculate personnel costs for the operations, ICU and peripheral ward based on the remuneration for each classification according to the collective agreements (TV-L and TV-Doctors University Hospital) for medical service and nursing service [34]. An employer contribution to social security (25.59%) was applied. Personnel costs were calculated at the case level using the collective agreement tariffs of the corresponding year (2020–2022). From that, means and standard deviations were calculated for each group using Excel 2019. Cost items such as pharmaceuticals or energy as well as personnel costs outside of medical treatment, for example cleaning or administration, were excluded.

OR personnel costs of each polytrauma case were calculated for all surgeons and surgical nurses involved in the operations according to the respective activity times (minutes) of the surgical protocols. As assumption, costs for an anesthesiologist and an anesthesia nurse for the entire duration of each surgical procedure were added.

Basically, ward and ICU personnel costs per case were derived from the costs per bed and 24-hour day multiplied by the length of stay, which implicitly assumes full occupancy.

For ICU and peripheral ward (each 24 beds), costs per day for a modeled staffing (medical and nursing service) were determined based on the assumptions for a University Hospital without universal validity. We used a 2-shift model for the medical staff in the ICU and a 1-shift model on the peripheral ward. Costs for nursing staff were each calculated using a 3-shift model, adhering to the regulations of minimum nursing staff levels in nursing-sensitive areas in hospitals. For peripheral ward, a patient-to nurse-ratio of 10:1 (day) and 20:1 (night), for ICU a ratio of 2:1 (day) and 3:1 (night) per shift was applied according to the Nursing Staff Lower Limits Ordinance (PpUGV).

Based on different medical and nursing staff on Monday-Friday and Saturday-Sunday/public holidays, we averaged personnel costs for ICU and peripheral ward for a 24-hour day over 7-day-week and calculated the proportional costs per bed/24-hour day by dividing the number of beds.

$$\:\mathrm{P}\mathrm{e}\mathrm{r}\mathrm{s}\mathrm{o}\mathrm{n}\mathrm{n}\mathrm{e}\mathrm{l}\:\mathrm{c}\mathrm{o}\mathrm{s}\mathrm{t}\mathrm{s}\:\mathrm{p}\mathrm{e}\mathrm{r}\:\mathrm{b}\mathrm{e}\mathrm{d}/\mathrm{d}\mathrm{a}\mathrm{y}=\:\frac{(\mathrm{C}\mathrm{o}\mathrm{s}\mathrm{t}\mathrm{s}\:\mathrm{n}\mathrm{u}\mathrm{r}\mathrm{s}\mathrm{i}\mathrm{n}\mathrm{g}\:\mathrm{s}\mathrm{e}\mathrm{r}\mathrm{v}\mathrm{i}\mathrm{c}\mathrm{e}+\mathrm{m}\mathrm{e}\mathrm{d}\mathrm{i}\mathrm{c}\mathrm{a}\mathrm{l}\:\mathrm{s}\mathrm{e}\mathrm{r}\mathrm{v}\mathrm{i}\mathrm{c}\mathrm{e})/\mathrm{d}\mathrm{a}\mathrm{y}\:}{24\:\mathrm{b}\mathrm{e}\mathrm{d}\mathrm{s}}$$

To determine personnel costs for ICU and peripheral ward per stay, personnel costs per bed/24-hour day were multiplied by the respective length of stay.

$$\begin{aligned} \mathrm{P}\mathrm{e}\mathrm{r}\mathrm{s}\mathrm{o}\mathrm{n}\mathrm{n}\mathrm{e}\mathrm{l}\:\mathrm{c}\mathrm{o}\mathrm{s}\mathrm{t}\mathrm{s}\:\mathrm{p}\mathrm{e}\mathrm{r}\:\mathrm{s}\mathrm{t}\mathrm{a}\mathrm{y}&=\mathrm{P}\mathrm{e}\mathrm{r}\mathrm{s}\mathrm{o}\mathrm{n}\mathrm{n}\mathrm{e}\mathrm{l}\:\mathrm{c}\mathrm{o}\mathrm{s}\mathrm{t}\mathrm{s}\:\mathrm{p}\mathrm{e}\mathrm{r}\:\mathrm{b}\mathrm{e}\mathrm{d}/\mathrm{d}\mathrm{a}\mathrm{y}\times\mathrm{L}\mathrm{O}\mathrm{S}\:\left(\mathrm{I}\mathrm{C}\mathrm{U}\:\mathrm{o}\mathrm{r}\:\mathrm{P}\mathrm{W}\right)\end{aligned}$$

Total ward personnel costs per inpatient stay were calculated by adding personnel costs for ICU and peripheral ward.

#### Surgery costs

Costs per operation were calculated by adding OR personnel costs and implant costs for each surgical intervention.

$$\:\mathrm{O}\mathrm{R}\:\mathrm{c}\mathrm{o}\mathrm{s}\mathrm{t}\mathrm{s}=\:\mathrm{O}\mathrm{R}\:\mathrm{p}\mathrm{e}\mathrm{r}\mathrm{s}\mathrm{o}\mathrm{n}\mathrm{n}\mathrm{e}\mathrm{l}\:\mathrm{c}\mathrm{o}\mathrm{s}\mathrm{t}\mathrm{s}+\mathrm{i}\mathrm{m}\mathrm{p}\mathrm{l}\mathrm{a}\mathrm{n}\mathrm{t}\:\mathrm{c}\mathrm{o}\mathrm{s}\mathrm{t}\mathrm{s}\:\:\:\:\:\:\:\:\:\:\:\:\:\:\:\:$$

Costs for all operations per group were added and means as well as standard deviations were calculated for group 1–11 and the total collective.

#### Contribution margins

To calculate contribution margins approximately, OR costs and modelled personnel costs for ICU and peripheral ward were deducted from the total revenues.

$$\begin{aligned} \mathrm{C}\mathrm{o}\mathrm{n}\mathrm{t}\mathrm{r}\mathrm{i}\mathrm{b}\mathrm{u}\mathrm{t}\mathrm{i}\mathrm{o}\mathrm{n}\:\mathrm{m}\mathrm{a}\mathrm{r}\mathrm{g}\mathrm{i}\mathrm{n}&=\mathrm{T}\mathrm{o}\mathrm{t}\mathrm{a}\mathrm{l}\:\mathrm{r}\mathrm{e}\mathrm{v}\mathrm{e}\mathrm{n}\mathrm{u}\mathrm{e}-[\mathrm{O}\mathrm{R}\:\mathrm{c}\mathrm{o}\mathrm{s}\mathrm{t}\mathrm{s}+(\mathrm{P}\mathrm{e}\mathrm{r}\mathrm{s}\mathrm{o}\mathrm{n}\mathrm{n}\mathrm{e}\mathrm{l}\:\mathrm{c}\mathrm{o}\mathrm{s}\mathrm{t}\mathrm{s}\:\mathrm{I}\mathrm{C}\mathrm{U}+\mathrm{P}\mathrm{W}\left)\right]\end{aligned}$$

It should be noted, that this is not a contribution margin in the usual sense, since relevant variable cost blocks (blood products, imaging and laboratory diagnostics, physiotherapy, non-implant consumables, emergency department and shock room) are not included, and these are systematically higher in the most severely injured groups. The calculated values represent an upper-bound estimate.

## Results

A total of 201 polytrauma cases were included with following distribution across the departments leading in polytrauma care at the University Hospital.

Anesthesiology: *n* = 13.

General Surgery: *n* = 16.

Neurosurgery: *n* = 44.

Trauma Surgery: *n* = 128.

**Fig. 2:**
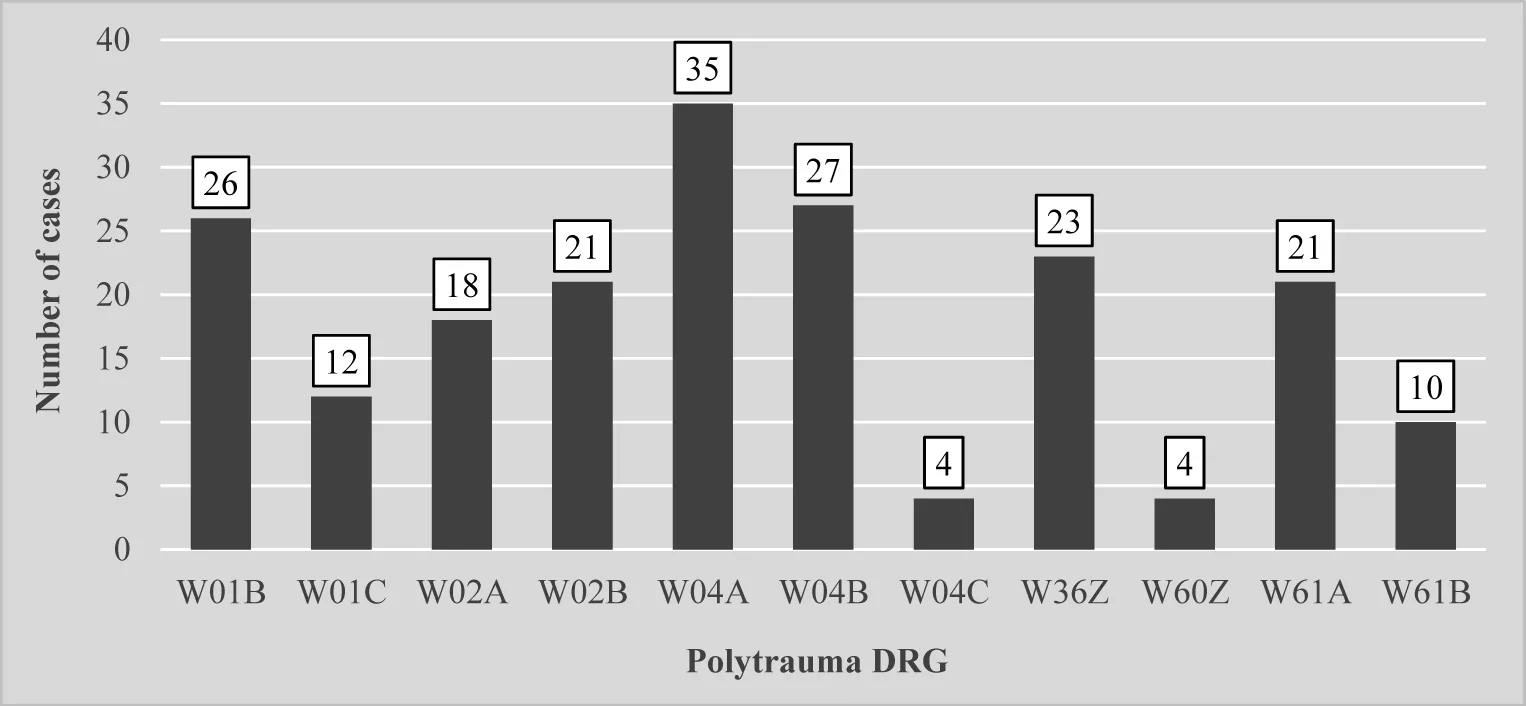
Case numbers per polytrauma DRG (Total number of cases: n = 201). DRG diagnosis related groups; n number

All cases were classified into polytrauma DRGs of MDC 21 A with different case numbers per DRG group (Fig. 2). DRG W04A (*n* = 35), W04B (*n* = 27) and W01B (*n* = 26) were the predominant ones, DRG W04C (*n* = 4) and W60Z (*n* = 4) the rarest ones in the entire collective.

**Fig. 3:**
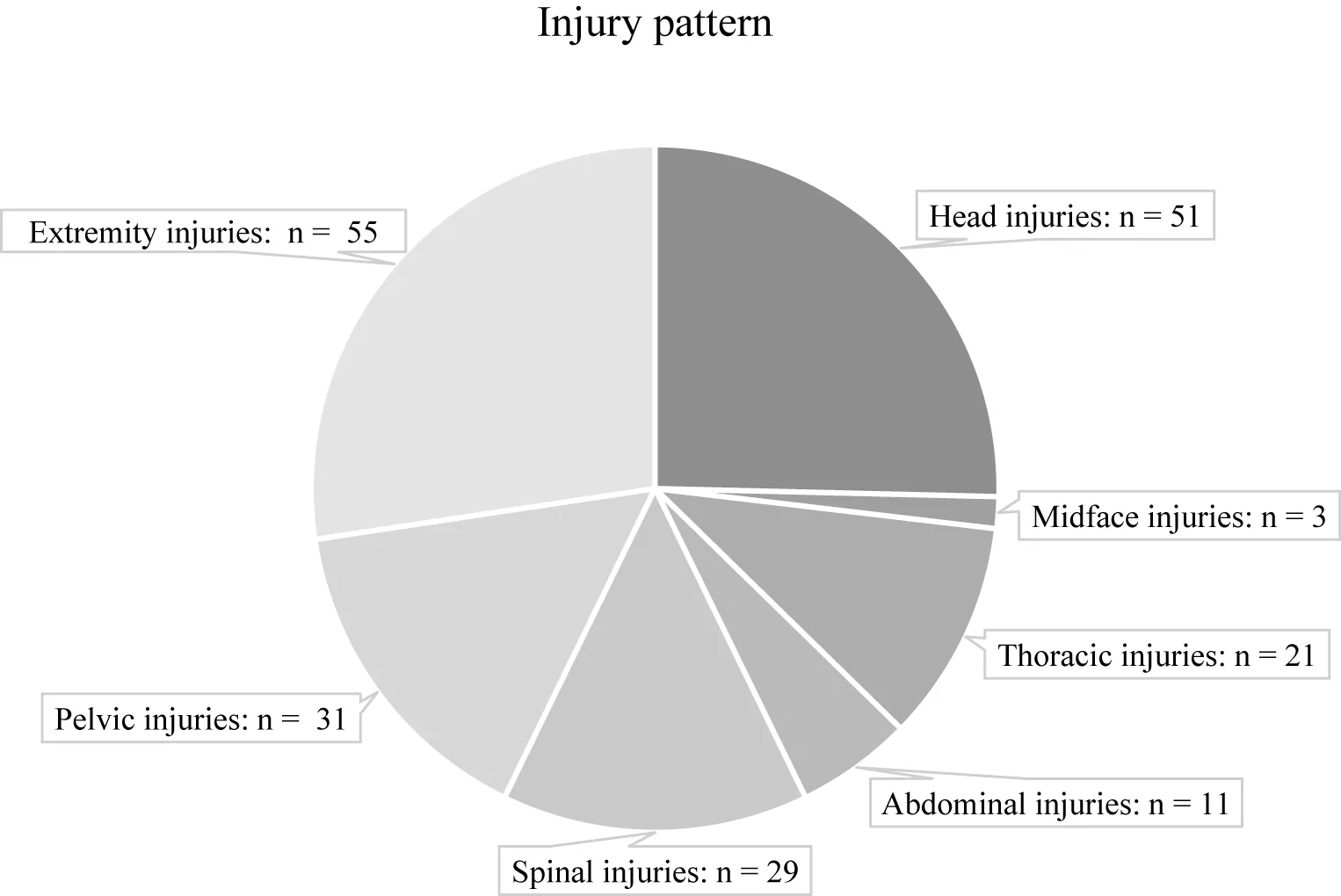
Injury pattern of the total collective (Only the leading injury is shown. Total number of cases: *n* = 201). *n* number

Fig. 3 shows the injury pattern of the total polytrauma collective derived only from the leading injury with the highest resource use, defined as main diagnosis in the G-DRG system. Leading injuries of the extremities (*n* = 55) and head (*n* = 51) were most frequent, followed by pelvic (*n* = 31) and spinal (*n* = 29) injuries.

Cases with dominant head injuries were mostly classified into DRG W01C, W36Z and W61A. Leading injuries of the midface were rare (*n* = 3) and grouped into DRG W01B, W04A and W36Z. Thoracic injuries (*n* = 21) were mainly found in DRG W04B and W61A, abdominal injuries (*n* = 11) in DRG W36Z and spinal injuries in DRG W01B but also W02A and W02B. Both pelvic and extremity injuries were predominantly assigned to DRG W04A and W04B.

### Total collective

70.1% of all polytrauma cases were male (141/201) and 29.9% were female (60/201) with an average age of 50.5 years. Mean ISS was 24.4 points. Total LOS in the hospital was approximately 17 days with an average LOS of seven days in the ICU and ten days on the peripheral ward. Mean OR time was 228.6 min and implant costs amounted to 2,446 €. The total revenue averaged 30,561 € including DRG revenue of 21,589 €, nursing care revenue of 8,118 € and additional charges of approximately 853 €. All parameters of the total collective calculated at the case level are presented in Table 2.

**Table 2:** Data of the total collective (Values shown are mean values with standard deviations in parentheses. For clearly skewed variables median and interquartile range are added)

Parameter	Total collective
DRG	W01B – W61B
Case number	201
Gender (female/male)	f: 60 / m: 141
Age (years)	50.5 (20.8)
ISS	24.4 (10.4)
PCCL	2.2 (1.8)
Total LOS (days)	16.9 (14.1)
LOS Peripheral Ward (days)	9.9 (10.0)
LOS ICU (days)	7.0 (8.6)
Ventilation time (hours)	74.4 (129.0) Md: 0; IQR: 80.0
OR time (minutes)	228.6 (238.9)
Deduction (€)	873.5 (2,294)
Surcharge (€)	991.1 (6,596)
Cost Weight	5.8 (4.3)
DRG revenue (€)	21,589 (16,301)
Care revenue (€)	8,118 (11,584)
Add. charge (€)	852.8 (2,816) Md: 0; IQR: 0
Total revenue (€)	30,561 (26,898)
Base rate (€)	3,726 (65.3)
Care fee value (€)	210.5 (79.2)
Implant costs (€)	2,446 (3,951) Md: 814.9; IQR: 2,971

Add. additional; DRG diagnosis related groups; IQR interquartile range; ISS injury severity score; ICU intensive care unit; LOS length of stay; Md median; OR operation; PCCL patient clinical complexity level

### Groups 1–11

The most severe polytraumas with an average ISS of 29 points, longest ICU stay and ventilation time were classified into DRG W36Z and DRG W01B. Less seriously injured patiens were assigned to DRG W04C and DRG W61B with mean rounded up ISS of 16 points. Severely injured patients who died within five days after admission to the hospital were assigned to DRG W60Z. Mean DRG revenue of 50,852 € was highest for DRG W36Z. Both OR time of 407.5 min and implant costs of 5,691 € were highest for DRG W02A. Table 3 provides an overview of all parameters for groups 1–11.

**Table 3:** Data for groups 1–11 (Values shown are mean values with standard deviations in parentheses. For clearly skewed variables median and interquartile range are added)

Parameter	Group 1	Group 2	Group 3	Group 4	Group 5	Group 6	Group 7	Group 8	Group 9	Group 10	Group 11
DRG	W01B	W01C	W02A	W02B	W04A	W04B	W04C	W36Z	W60Z	W61A	W61B
Case number	26	12	18	21	35	27	4	23	4	21	10
Gender (f/m)	f: 6 / m: 20	f: 5 / m: 7	f: 6 / m: 12	f: 4 / m: 17	f: 12 / m: 23	f: 5 / m: 22	f: 3 / m: 1	f: 6 / m: 17	f: 2 / m: 2	f: 7 / m: 14	f: 4 / m: 6
Age (years)	51.4 (22.6)	51.0 (22.3)	49.1 (19.7)	44.0 (15.4)	47.1 (21.9)	46.4 (19.9)	69.0 (12.7)	53.4 (20.0)	61.8 (27.7)	56.8 (18.6)	55.0 (17.9)
ISS	28.6 (9.8)	26.1 (8.3)	29.0 (9.2)	19.4 (7.8)	25.9 (12.4)	20.3 (8.2)	16.3 (5.8)	29.0 (9.7)	39.3 (3.4)	20.0 (6.7)	15.6 (4.7)
PCCL	3.5 (1.3)	2.5 (1.4)	3.0 (1.8)	1.8 (1.5)	2.2 (1.8)	1.2 (1.4)	1.8 (1.3)	4.1 (1.2)	1.8 (1.1)	0.8 (1.0)	0.3 (0.5)
Total LOS (days)	25.4 (12.1)	13.9 (7.4)	19.9 (10.1)	12.2 (6.4)	14.7 (12.6)	12.2 (7.0)	16.3 (1.5)	34.0 (22.5)	1.0 (0)	9.3 (5.4)	7.2 (3.6)
LOS PW (days)	9.3 (11.4)	7.6 (5.5)	14.5 (9.1)	9.5 (6.7)	11.5 (11.8)	10.3 (7.0)	13.5 (2.6)	11.0 (16.5)	0.5 (0.5)	7.2 (4.0)	6.4 (3.6)
LOS ICU (days)	16.2 (4.2)	6.3 (4.7)	5.4 (3.3)	2.7 (2.1)	3.3 (3.5)	1.9 (2.4)	2.8 (2.6)	23.0 (9.6)	0.5 (0.5)	2.1 (2.2)	0.8 (0.9)
Ventilation time (h)	234.3 (133.4)	32.8 (39.3)	29.7 (42.7)	4.8 (16.4)	23.0 (36.6)	4.1 (11.4)	8.0 (9.8)	298.5 (126.0)	2.8 (3.3)	0.95 (4.0)	0 (0)
	Md: 225.0	Md: 16.0	Md: 6.0	Md: 0	Md: 0	Md: 0	Md: 4.0	Md: 313.0	Md: 1.5	Md: 0	Md: 0
	IQR: 177.3	IQR: 45.3	IQR: 41.3	IQR: 0	IQR: 33.5	IQR: 0	IQR: 12.0	IQR: 188.0	IQR: 4.3	IQR: 0	IQR: 0
OR time (min)	381.8 (244.3)	175.2 (164.5)	407.5 (206.7)	248.5 (102.7)	264.1 (240.5)	114.8 (93.8)	57.8 (19.8)	381.1 (312.7)	0 (0)	0 (0)	2.0 (4.0)
Deduction (€)	0 (0)	1,136 (2,681)	1,418 (3,724)	1,498 (2,504)	2,134 (3,183)	836.9 (1,733)	0 (0)	0 (0)	0 (0)	227.6 (1,018)	287.9 (863.8)
Surcharge (€)	1,073 (3,720)	0 (0)	0 (0)	0 (0)	478.6 (2,791)	12.3 (62.9)	0 (0)	6,636 (17,762)	0 (0)	77.6 (347.2)	0 (0)
Cost Weight	10.6 (1.0)	3.8 (0.8)	7.5 (1.0)	4.2 (0.7)	4.9 (1.2)	2.9 (0.5)	2.37 (0)	13.6 (4.8)	0.6 (0)	1.7 (0.3)	1.1 (0.2)
DRG revenue (€)	39,433 (3,794)	14,019 (2,882)	27,873 (3,814)	15,475 (2,510)	18,281 (4,565)	10,950 (1,671)	8,953 (75.4)	50,852 (18,512)	2,214 (121.3)	6,142 (1,103)	4,056 (850.7)
Care revenue (€)	14,310 (8,488)	7,932 (6,841)	5,646 (3,000)	3,323 (2,638)	5,439 (7,644)	3,406 (2,289)	4,409 (1,751)	26,407 (21,330)	272.3 (30.0)	3,218 (3,197)	1,715 (572.0)
Add. charge (€)	2,066 (4,799)	995.4 (1,759)	1,587 (4,135)	0 (0)	581.6 (1,873)	116.7 (310.9)	0 (0)	2,333 (3,988)	0 (0)	0 (0)	0 (0)
	Md: 0	Md: 0	Md: 0	Md: 0	Md: 0	Md: 0	Md: 0	Md: 0	Md: 0	Md: 0	Md: 0
	IQR: 674.8	IQR: 813.1	IQR: 624.7	IQR: 0	IQR: 0	IQR: 0	IQR: 0	IQR: 2,111	IQR: 0	IQR: 0	IQR: 0
Total revenue (€)	55,809 (12,593)	22,947 (8,291)	35,106 (3,520)	18,798 (4,455)	24,302 (11,342)	14,472 (3,530)	13,362 (1,709)	79,593 (36,442)	2,487 (151.0)	9,360 (3,795)	5,771 (1,027)
Base rate (€)	3,729 (63.3)	3,708 (62.0)	3,710 (61.9)	3,703 (65.0)	3,737 (67.9)	3,741 (59.6)	3,782 (43.1)	3,728 (66.9)	3,681 (34.0)	3,730 (68.4)	3,717 (51.8)
Care fee value (€)	202.1 (64.4)	219.7 (85.7)	175.8 (16.6)	188.8 (58.9)	207.7 (68.0)	232.1 (92.3)	234.3 (82.9)	224.8 (108.6)	179.5 (9.5)	215.9 (84.5)	239.2 (89.9)
Implant costs (€)	2,843 (2,604)	714.3 (1,046)	5,691 (3,760)	2,246 (1,360)	3,020 (3,994)	1,552 (3,680)	332.1 (115.4)	4,808 (6,954)	0 (0)	0 (0)	0 (0)
	Md: 2,028	Md: 461.6	Md: 6,523	Md: 2,216	Md: 1,322	Md: 143.6	Md: 391.9	Md: 1,347	Md: 0	Md: 0	Md: 0
	IQR: 2,513	IQR: 304.7	IQR: 6,722	IQR: 1,830	IQR: 3,526	IQR: 1,156	IQR: 69.8	IQR: 6,945	IQR: 0	IQR: 0	IQR: 0

Add. additional; DRG diagnosis related groups; f female; h hours; ICU intensive care unit; IQR interquartile range; ISS injury severity score; LOS length of stay; m male; Md median; min minutes; OR operation; PCCL patient clinical complexity level; PW peripheral ward

### Ratios

Revenue in relation to total LOS (Ratio 1) was highest for group 9 (2,487 €/day, DRG W60Z) and lowest for group 11 (802 €/day, DRG W61B). With regard to LOS in ICU (Ratio 2), the revenue of 7,657 €/day was highest for group 6 (DRG W04B), lowest for group 8 (3,455 €/day, DRG W36Z). Group 8 showed the highest Ratio 3 of 7,236 €/day (DRG W36Z), group 11 the lowest one (902 €/day, DRG W61B). Ratio 4 was highest for group 4 (3,949 €/hour, DRG W02B) and lowest for group 1 (238 €/hour, DRG W01B). DRG revenue of 155 € per OR minute (Ratio 5) was highest in group 7 (DRG W04C) and lowest in group 4 (62 €/minute, DRG W02B). Regarding implant costs (Ratio 6), the revenue of 30 € per euro of implant costs was highest for group 7 (DRG W04C), lowest for group 3 (5 € per euro of implant costs, DRG W02A). Since the value of Ratio 4 in group 10 results from a mean ventilation time of approximately one hour, and the value of Ratio 5 for group 11 from a mean OR time of two minutes, they are not interpretable (n.a.). Where the denominator is zero (no ventilation in group 11, no surgery in groups 9 and 10, no implants in groups 9 to 11), the ratio is undefined (n.a.).

**Table 4:** Ratios for groups 1–11 and the total collective

Groups and total collective	Ratio 1 (€/day)	Ratio 2 (€/day)	Ratio 3 (€/day)	Ratio 4 (€/hour)	Ratio 5 (€/minute)	Ratio 6 (€/€)
1: W01B 2: W01C 3: W02A 4: W02B 5: W04A 6: W04B 7: W04C 8: W36Z 9: W60Z 10: W61A 11: W61B Total collective	2,195 1,648 1,761 1,542 1,652 1,187 822 2,338 2,487 1,008 802 1,808	3,456 3,625 6,453 7,040 7,455 7,657 4,859 3,455 4,973 4,457 7,214 4,366	6,020 3,027 2,421 1,975 2,121 1,405 990 7,236 4,973 1,302 902 3,087	238 701 1,181 3,949 1,058 3,521 1,670 267 904 n.a. n.a. 411	103 80 68 62 69 95 155 133 n.a. n.a. n.a. 94	14 20 5 7 6 7 30 11 n.a. n.a. n.a. 9

Ratio 1: Total revenue/Total LOS (€/day) Ratio 2: Total revenue/LOS ICU (€/day) Ratio 3: Total revenue/LOS peripheral ward (€/day) Ratio 4: Total revenue/Ventilation time (€/hour) Ratio 5: DRG revenue/ OR time (€/minute) Ratio 6: DRG revenue/implant costs (€/€ = revenue per euro of implant costs) Ratios 1–4 were calculated from total revenue (including DRG revenue, nursing care revenue and additional charges). Ratios 5 + 6 were calculated from DRG revenue

### Surgery costs

Total costs per operation of 8,415 € were highest for group 3 (DRG W02A) and lowest for group 11 (DRG W61B) with approximately 5 €. For the total collective, mean total costs per operation of 3,886 € were calculated (Table 5).

**Table 5:** Cost calculation per operation for groups 1–11 and the total collective (Values shown are mean values with standard deviations in parentheses)

Costs per operation (€)			
Groups and total collective	Implant costs (€)	Personnel costs (€)	Total costs (€)
1: W01B 2: W01C 3: W02A 4: W02B 5: W04A 6: W04B 7: W04C 8: W36Z 9: W60Z 10: W61A 11: W61B Total collective	2,843 (2,604) 714.3 (1,046) 5,691 (3,760) 2,246 (1,360) 3,020 (3,994) 1,552 (3,680) 332.1 (115.4) 4,808 (6,954) 0 (0) 0 (0) 0 (0) 2,446 (3,951)	2,432 (1,682) 931.1 (905.4) 2,724 (1,609) 1,613 (923.1) 1,629 (1,549) 671.3 (590.4) 323.1 (108.3) 2,420 (2,165) 0 (0) 0 (0) 4.9 (9.9) 1,440 (1,616)	5,275 1,645 8,415 3,859 4,649 2,223 655.2 7,228 0 0 4.9 3,886

### Personnel costs

For the ICU, highest personnel costs were calculated for group 8 (DRG W36Z, 17,572 €) and minimum costs of 376.2 € for group 9 (DRG W60Z). The averaged costs on peripheral ward were highest for group 3 (DRG W02A, 2,633 €) and lowest for group 9 (DRG W60Z, 90.5 €). Total personnel costs (ICU + peripheral ward) of 19,590 € were highest for group 8 (DRG W36Z) and lowest for group 9 (DRG W60Z, 466.7 €). For the entire collective, total ward personnel costs of 7,160 € were calculated (Table 6).

**Table 6:** Calculation of personnel costs peripheral ward, ICU and total costs (ICU + peripheral ward) for groups 1–11 and the total collective (Values shown are mean values with standard deviations in parentheses)

Personnel Costs (€)			
Groups and total collective	Intensive Care Unit (€)	Peripheral Ward (€)	ICU + PW (€)
1: W01B 2: W01C 3: W02A 4: W02B 5: W04A 6: W04B 7: W04C 8: W36Z 9: W60Z 10: W61A 11: W61B Total collective	12,334 (3,292) 4,834 (3,589) 4,137 (2,491) 2,019 (1,554) 2,490 (2,658) 1,448 (1,876) 2,106 (1,973) 17,572 (7,352) 376.2 (376.2) 1,598 (1,708) 608.6 (662.6) 5,351 (6,538)	1,697 (2,087) 1,372 (987.1) 2,633 (1,647) 1,724 (1,210) 2,090 (2,141) 1,876 (1,268) 2,480 (479.9) 2,018 (3,041) 90.5 (90.5) 1,308 (734.0) 1,158 (650.9) 1,809 (1,839)	14,031 6,206 6,770 3,743 4,580 3,324 4,586 19,590 466.7 2,906 1,766 7,160

ICU intensive care unit; PW peripheral ward

### Contribution margins

Table 7 shows the approximated contribution margins as an upper-bound estimate with highest value of 52,775 € for group 8 (DRG W36Z) and lowest value of 2,021 € for group 9 (DRG W60Z). For the total collective, a mean value of 19,515 € was calculated.

**Table 7:** Contribution margins for groups 1–11 and the total collective (Values shown represent an upper-bound estimate)

Groups and total collective	Total revenue (€)	OR costs (€)	Personnel costs ICU + PW (€)	Contribution margin (€)
1: W01B 2: W01C 3: W02A 4: W02B 5: W04A 6: W04B 7: W04C 8: W36Z 9: W60Z 10: W61A 11: W61B Total collective	55,809 22,947 35,106 18,798 24,302 14,472 13,362 79,593 2,487 9,360 5,771 30,561	5,275 1,645 8,415 3,859 4,649 2,223 655.2 7,228 0 0 4.9 3,886	14,031 6,206 6,770 3,743 4,580 3,324 4,586 19,590 466.7 2,906 1,766 7,160	36,503 15,096 19,921 11,196 15,073 8,925 8,121 52,775 2,021 6,454 4,000 19,515

ICU intensive care unit; OR operation; PW peripheral ward

## Discussion

The purpose of this study was to analyze DRG revenues and costs for the eleven polytrauma DRGs of MDC 21 A with regard to length of stay, ventilation and OR time, implant costs and modelled personnel costs in the current German payment system.

First, it should be noted that the study period 2020 to 2022 coincides with the COVID-19 pandemic and the first years after the nursing budget carve out. Both plausibly influenced case numbers, ICU capacity, length of stay and the revenue structure.

As main finding of the study, both DRG revenue and the approximated contribution margin were highest for the most severely injured patients (52,775 € for DRG W36Z and 36,503 € for DRG W01B), while revenue per ICU day and per ventilation hour was lowest in exactly these groups. A high absolute margin can coexist with poor coverage of the resources actually consumed, and whether the margin is truly positive depends on the cost blocks that could not be analyzed here.

Another important finding concerns DRG revenue in relation to OR time and implant costs. For severely injured patients with predominantly injuries and multiple surgical interventions of the extremities, spine and pelvis (DRG W02A and DRG W02B), DRG revenue per OR minute and per implant costs was lowest due to long OR times and costly implants used.

The majority of the 201 polytrauma cases included were male (*n* = 141) with an average age of 50.5 years and thus in an age group that is occupationally active. Due to the long recovery period and lost work time, the treatment of severely injured patients is associated with high overall treatment costs and serious economic effects as Anders et al. showed by empirical analysis of the direct, indirect and intangible costs of polytraumatized patients [8]. In our results, the mean ISS of the total collective was 24.4 points, so that by definition they were severely injured (ISS ≥ 16) and their classification into MDC 21 A DRGs was appropriate. Using cost analysis of trauma patients in the DRG system, Garving et al. demonstrated that an increase in injury severity classified according to ISS is associated with rising treatment costs and decreasing contribution margins [35]. Mabry et al. also found an association between increasing ISS and higher costs by determining polytrauma care costs for US trauma centers [36].

Besides the injury pattern, other factors like surgical strategies, DCO or Safe Definitive Surgery certainly also have an impact on polytrauma care, length of hospital stay and treatment costs. In a way, this is reflected by the corresponding DRG classification of polytrauma cases that our revenue and cost analyses were based on. However, this study did not aim to analyze the impact of these relevant factors in the context of polytrauma reimbursements and costs in detail.

In our study, the DRG revenue of the entire collective amounted to 21,589 € and the total revenue, including 26.6% nursing care reimbursements and 2.8% additional charges, to 30,561 €. Compared to the findings reported by Schopow et al. [13] the total revenue is higher. The authors determined a total revenue of 24,120 € (including 2.3% additional charges and 24.5% nursing care reimbursements) for “DRG-Polytrauma” by cost-benefit analysis of polytrauma care within the aG-DRG system using a modified cost model according to Pape et al. [19].

Analyzing groups 1–11, DRG revenues of 50,852 € and 39,433 € respectively were highest for the most seriously injured patients assigned to DRG W36Z and DRG W01B. Mainly polytrauma cases with leading severe head injuries or multiple injuries of the extremities, long LOS in the hospital (≥ 25 days) with complex ICU treatment (≥ 16 days), long ventilation time (≥ 234 h) and multiple surgical interventions with OR time up to 382 min were classified here.

DRG revenue of 2,214 € was lowest for multiple injured patients deceased within five days after admission to the hospital (DRG W60Z). Polytrauma cases classified into DRG W61B without significant surgical interventions or ventilation and short total LOS of approximately seven days showed second lowest DRG revenue of 4,056 €. Since these groups are very small and means derived from four and ten cases respectively, the results should be interpreted with great caution.

The average total LOS in the hospital of the entire collective was approximately 17 days with mean ICU treatment of seven days and LOS of ten days on the peripheral ward. Comparing the eleven groups, DRG revenue in relation to total LOS of 2,487 € was highest for DRG W60Z, since patients died shortly after admission. However, DRG revenue of 2,338 € per day was second highest for the most severely injured patients (DRG W36Z) despite the longest stay in the hospital of 34 days due to the highest total revenue.

An analysis of predictors for higher treatment costs of trauma from Curtis et al. showed an association between LOS but also admission to the ICU and increasing costs for trauma centers [37]. Fountain et al. concluded that extensive length of stay in the treatment of patients with traumatic brain injury is associated with financial losses for the hospitals [38]. Further national and international studies reported a correlation between LOS and ICU stay and an increase of the overall treatment costs in care of severely injured patients [39, 40]. Fakhry et al. concluded that LOS of trauma patients correlates with profitability of the hospitals and identified a LOS of eleven days as “inflection point” defining the profitable patient`s cohort [41].

Besides the severity of the injuries and necessary therapeutic measures, including adequate and sufficient mobilisation of the patients, also other structural factors regarding further care can influence LOS in the hospitals and thus treatment costs. Jukema et al. showed by single-centre case study under Swiss DRG terms that an extension of LOS by 5.1 days due to waiting for transfer to rehabilitation facility caused a financial burden of 885,360 Swiss francs without reimbursement for the hospital [42].

Our results showed the lowest revenues of 3,455 € and 3,456 € in relation to LOS in the ICU for the most severely injured patients (DRG W36Z and DRG W01B) despite the highest total revenues due to complex and long ICU treatment up to 23 days. Revenue per ventilation hour (238 € and 267 €) was also lowest for the most seriously injured groups, since patients had the longest ventilation times.

High ICU treatment efforts are very costly and cause together with the surgical interventions the majority of total costs in polytrauma care [10, 43, 44]. Dasta et al. concluded that costs are highest within the first two days after admission to the intensive care unit and that longer ICU stay as well as extended mechanical ventilation is associated with significantly higher daily costs for intensive care treatment [45]. An analysis of the TraumaRegister DGU^®^ from Böhmer et al. showed that LOS in ICU was reduced from approximately twelve to nine days in recent years, which was influenced by the lower rate of intubated patients when admitted to ICU [46]. ICU care in the treatment of most severely injured patients can lead to financial losses for trauma centers particularly with regard to extreme cost intensive cases, as Hebler et al. demonstrated [47].

Concerning surgical treatments in the present study, DRG revenue of 62 € in relation to OR minute was lowest for patients with multiple surgical interventions of the spine and pelvis (DRG W02B). Due to the longest OR time of 407.5 min, DRG revenue of 68 € was second lowest for higher classified cases (DRG W02A) with leading injuries of the extremities. Different studies identified long operative time and high number of surgical interventions as predictors for increasing treatment costs and financial losses in the care of severely injured patients [48–50]. Besides OR time, implant costs can be main cost drivers in polytrauma care. In our study, DRG W02A showed the lowest revenue of 5 € per euro of implant costs due to the highest average costs of 5,691 € for implants mainly used for operations on the extremities. A correlation between injuries of the extremities with need for surgical interventions and increasing costs but also financial losses for a Swiss trauma center was demonstrated by Gross et al. [51].

As mentioned at the beginning, the approximated contribution margins of 52,775 € and 36,503 € were highest for the most severely injured patients in our study. Since it can be assumed that overall treatment costs for exactly these cases are the highest with poor coverage, this represents an apparent contradiction. However, these are merely approximately determined contribution margins, which do not allow any conclusions about cost coverage rate of polytrauma care and profitability of the University Hospital. An analysis of further cost blocks, such as emergency department infrastructure or standby capacity of OR and ICU, was not possible due to structural reasons regarding cost recording at the hospital.

Generally, treatment of severely injured patients in specialized trauma centers requires 24/7 availability of shock room, OR and ICU resources, which is associated with high readiness costs and serious economic challenges, as different studies demonstrated [52, 53]. Our data suggest that the resource use in the treatment of the most severely injured patients is not adequately reflected by case-based reimbursement alone, which supports the concept of separate readiness payments as currently discussed within the German hospital reform [21, 22]. According to the current situation, readiness payments are supposed to be introduced from 2028 and distributed to the hospitals via assigned special performance groups [54].

## Limitations

This study is limited by the fact that an analysis of total treatment costs per case was not possible due to missing cost data because of the special cost recording structure at the University Hospital. Therefore, no final conclusion is possible, whether the revenues for polytrauma care are cost-covering or result in financial losses. Furthermore, personnel costs were calculated by model and contribution margins merely approximated as an upper-bound estimate, which do not allow any conclusions about cost coverage rate and profitability of the hospital. Another limiting fact concerns the generalizability of the results, as the calculations of personnel costs and approximations of contribution margins are not universally applicable rather hospital-specific outputs. Personnel costs as well as implant costs are varying portions of treatment costs influencing cost coverage rate.

DRG-based case selection and the very small case numbers in some groups are further limiting factors. The fact that the calculations are based on the base rate of a single federal state and the care fee value of a single hospital limits transferability. Another limitation is the absence of any case-mix or comorbidity adjustment, so that differences between the DRG groups also reflect differences in patient characteristics rather than in reimbursement logic alone. This study is also limited by the purely descriptive statistical analysis with missing formal statistical tests.

## Conclusions

Treatment of polytrauma patients with long ICU stay, extended mechanical ventilation and multiple injuries of the extremities, pelvis and spine is not adequately reflected by the current per-case reimbursement in relation to the resources used (ICU days, ventilation hours, OR time and implant costs). Since merely revenues and no total treatment costs were analyzed in this study, a final profit-loss calculation from the hospital`s perspective is not possible. However, seeking further improvements to the remuneration structure for polytrauma care in the German health care system seems justified.

## Supplementary Information

Below is the link to the electronic supplementary material.

Supplementary Material 1

## Data Availability

The datasets generated and analyzed during the current study are available from the corresponding author upon reasonable request. All data are located in controlled access data storage at the University Hospital Regensburg.
