# Supplementary material for: Health economic evaluation of polytrauma treatment in the current aG-DRG system – Results of a Level I Trauma Center
Source: Eur J Trauma Emerg Surg. 2026 Oct 23;52(1):329. doi: 10.1007/s00068-026-03368-1 (PMC13694449; doi:10.1007/s00068-026-03368-1)
Supplement: Supplementary file 1 [file 68_2026_3368_MOESM1_ESM.docx]

#### Keywords

Multiple trauma

Injury Severity Score

Operating Room

Intensive Care Unit

Reimbursement

Cost calculation
